# The relationship between movement speed and duration during soccer matches

**DOI:** 10.1371/journal.pone.0181781

**Published:** 2017-07-25

**Authors:** Kai Roecker, Hubert Mahler, Christian Heyde, Mareike Röll, Albert Gollhofer

**Affiliations:** 1 Furtwangen University, Applied Public Health (AGW), Furtwangen, Germany; 2 Albert-Ludwigs-University Freiburg, Institute of Sports and Sports Science (IfSS), Freiburg im Breisgau, Germany; 3 adidas AG, Future Team, Herzogenaurach, Germany; Sao Paulo State University, BRAZIL

## Abstract

The relationship between the time duration of movement (t(dur)) and related maximum possible power output has been studied and modeled under many conditions. Inspired by the so-called power profiles known for discontinuous endurance sports like cycling, and the critical power concept of Monod and Scherrer, the aim of this study was to evaluate the numerical characteristics of the function between maximum horizontal movement velocity (HSpeed) and t(dur) in soccer. To evaluate this relationship, GPS data from 38 healthy soccer players and 82 game participations (≥30 min active playtime) were used to select maximum HSpeed for 21 distinct t(dur) values (between 0.3 s and 2,700 s) based on moving medians with an incremental t(dur) window-size. As a result, the relationship between HSpeed and Log(t(dur)) appeared reproducibly as a sigmoidal decay function, and could be fitted to a five-parameter equation with upper and lower asymptotes, and an inflection point, power and decrease rate. Thus, the first three parameters described individual characteristics if evaluated using mixed-model analysis. This study shows for the first time the general numerical relationship between t(dur) and HSpeed in soccer games. In contrast to former descriptions that have evaluated speed against power, HSpeed against t(dur) always yields a sigmoidal shape with a new upper asymptote. The evaluated curve fit potentially describes the maximum moving speed of individual players during the game, and allows for concise interpretations of the functional state of team sports athletes.

## Introduction

Metabolic, locomotor, and physical performance factors such as maximum sprint speed, endurance, or repeated sprint ability [1] contribute to the success of players in team sports such as soccer (association football). These factors can be observed as locomotor events using a relatively new type of wearable measurement device comprising high-resolution GPS combined with accelerometers and/or gyroscopes [2,3]. These micro-sensors not only continually measure GPS locations and thus running speeds but also, through the use of accelerometers and gyroscopes, register complex body movement dynamics [4,5].

However, uncategorized time-ordered motion data from players of soccer and other team sports are generally chaotic and not obviously interpretable, nor even functionally describable, between repeated samples [6–8]. Thus, the locomotor activities performed during soccer matches remain difficult to correctly classify into categories and are not clearly interpretable by either coaches or athletes.

Most past efforts to classify sports-related movements have used automated or semi-automated visual video analyses [9]; thus, fixed velocity limits or classifications by comparative analysis of distinct locomotion types (e.g., standing, walking, jogging, and sprinting) have been proposed for the interpretation of sports-specific metabolic or locomotor demands [10–14]. However, recently researchers have increasingly used so-called wearable tracking devices, and have mainly focused on factors such as cumulative running distances within classified speed categories [14–17], tactical maneuvers, or specific metabolic load indices for locomotor activities, such as "Player Load" [18,19] or "Metabolic Power" [8,20].

At present, no distinct and automatically applicable algorithm has been established that can compare the locomotor profiles of individual players during matches. Arbitrary time intervals are currently used to analyze player performance during sports games (e.g., 5 min for high intensity) [21], but there is no approach that describes these requirements in an holistic and robust way. We therefore propose a derivation of locomotor analysis that enables the direct comparison and performance diagnostics of whole match plays from computerized data preparation based on the principle of power-to-duration profiles as formerly shown for bicycling [22,23]. Beyond such graphic depictions, we further aimed to select and evaluate a numerical fitting model for the description of these types of profiles.

Following approaches introduced to derive maximum power outputs from discontinuous endurance competitions such as bicycling or mountain bike racing, we previously argued that competitors would present their individual power maxima at least *once* during a cycling competition. Clustering all maximum power outputs measured by force-sensitive cranks to the specified time domains, individual maximum “power profiles” or envelope-curves were generated, characterizing the individual performances of the related cyclists [22].

The aim of this study was to evaluate the relationship between effective power output (horizontal movement velocity, *HSpeed*) and the time duration of movement (*t*_*dur*_) during soccer games. Thus, this study should evaluate whether there is a general and unique function that describes the relationship between *HSpeed* and *t*_*dur*_ in soccer. Such a general function describing the performance/power output of soccer players would be a powerful tool that could be used to improve the individual assessment of the distribution of metabolic and conditional preconditions in soccer. As a result, this kind of working model would allow analysis of "chaotic" data from soccer matches and would make it much easier for coaches and athletes to interpret individual performances.

## Materials and methods

### Participants

Thirty-eight healthy members of three soccer teams participated in this study. The first team (team A) played in the 5th German men’s amateur soccer league ("Oberliga"; 13 male participants: age 24.4 ± 2.4 y, body weight 78.6 ± 4.2 kg, height 181 ± 5 cm), the second team (team B) in the 4th German women's soccer league ("Oberliga"; 12 female participants: age 18.7 ± 3.3 y, weight 59.6 ± 6.9 kg, height 167 ± 2 cm), and the third team (team C) in the 8th German men’s amateur soccer league ("Kreisliga"; 13 male participants, age 22.6 ± 3.4 y, weight 75.3 ± 6.7 kg, height 182 ± 4 cm). The mean age, weight and height of team B was significantly (p < 0.01) lower than those of teams A and C. The positions played by the study participants are listed in Table 1.

**Table 1:** Players' positions.

	Position					
Team (No. of Games)	Central Defender	Central Midfielder	External Midfielder	Full-Back	Striker	All
**A** (3)	2	3	4	3	1	13
**B** (2)	2	3	5	3	3	16
**C** (3)	2	5	2	2	3	14
**All**	6	11	11	8	7	43

Number of players analyzed at each playing position.

The study protocol was approved by the ethics committee of Albert-Ludwigs-University Freiburg in accordance with the latest revision of the Declaration of Helsinki. All participants gave their written informed consent following full disclosure of the study protocol and procedures.

### Motion analysis

#### Sensors

Each participant wore a wearable tracking device (miCoach Elite Team System, adidas, Herzogenaurach, Germany) during the games, which was fixed into position on the back between the shoulder blades. The sensor location was chosen to maximize the validity of the recordings without hiding the athlete's body center, to ensure a sufficient connection with the global positioning system (GPS), and to minimize disturbances to the player during activity. The device contained a 10-Hz (10 samples per second) global positioning system (GPS) combined with an inertial measurement unit.

#### Data acquisition

The devices were applied 45 min before kick-off and were worn until 30 min after the end of each game by all field players except the goal keepers. Data recorded before kick-off and after the final whistle were discarded prior to analysis. All other data, including movements during the half-time break and during any game interruptions by the referee during regular play time, remained within the analysis. Games from players with < 45 min play time were excluded from the calculations of the speed to movement duration relationships.

#### Computations

First, timed tracking points from the GPS were converted to distances and filtered through a Kalman filter [24] running at 33 Hz, converting the player-centric frame of reference of the data to a fixed frame of reference with respect to the earth’s surface (axes: East, North and Up). The outputs from the filter algorithm were then used to report and save the filtered location, *HSpeed*, and accumulated running distances at 10 Hz.

To analyze the basic relationship between *t*_*dur*_ and *HSpeed* for each player and game, we applied so-called "moving medians". Medians were preferred over (moving) arithmetic means, because medians often give superior results due to their stability against outliers [25]. Hence, a sequence of moving medians of *HSpeed* against the course of play time (*t)* were computed (Eqs 1 and 2):

$$HSp\tilde{eed}{\left(t_{i}^{*}\right)}_{j}=median\left(HSpeed(t_{i}-t_{dur}{}_{j}/2),\cdots ,HSpeed(t_{i}+t_{dur}{}_{j}/2)\right)$$ (1)

where (Eq 2)

$$\begin{array}{ll} t_{i} & =t_{dur}{}_{j}/2+0.1s,\cdots ,T-t_{dur}{}_{j}/2\\ \text{j} & =\;1,\cdots ,21 \end{array}$$ (2)

*T* is the total time recorded in each experiment, and *t*_*dur*_ is an ordered set of 21 unique time durations (*t*_*durj*_) determining the window sizes for the median calculations (Eq 3):

$$t_{dur}=\;\left\{0.3,0.5,1,2,3,4,5,6.5,10,13.5,18,30,60,120,300,600,900,1200,1800,2400,2700\text{s}\right\}$$ (3)

These procedures resulted in 21 distinct time-ordered sets of median-smoothed *HSpeed*, one for every time duration domain *t*_*durj*_ and for every player and game. Algorithms proposed by Härdle and Steiger [26] were used to increase computational efficiency and to optimize program running times for these computations.

From every median smoothed set $HSp\tilde{eed}{\left(t_{i}^{\text{*}}\right)}_{j}$, unique maximum values for every *t*_*durj*_ were selected, resulting in distinctive profiles of maximum *HSpeed ($HSpeed_{max_{j}}$)* for any specific time duration *t*_*durj*_ of the mentioned set (Eq 4)):

$$HSpeed_{max_{j}}=\begin{array}{c} \begin{array}{c} {\text{max}}_{i\;\in \;n_{j}}\left(HSpeed{\left(t_{i}\right)}_{j}\right) \end{array} \end{array}$$ (4)

Here, *n*_*j*_ is the size of the related median-smoothed data set. For every player and every game, the resulting set contains 21 $HSpeed_{max_{j}}$ values, one for each member of the ordered set *t*_*durj*_.

Soccer-specific movement characteristics might not only depend on maximum moving speeds, but also on agility [27], which is potentially represented by dynamic changes in *HSpeed* over time. Therefore, we additionally calculated moving medians [28] weighted with the absolute values of concurrent horizontal acceleration (|*HAccel*|). Thus, the value pairs (*HSpeed*(*t*_*i*_), |*HAccel*|(*t*_*i*_)) in each of the time windows described in Eqs 1 to 3 were transformed into ascending order by *HSpeed*(*t*_*i*_). In each set, the weighted median corresponds to the element *HSpeed*(*t*_*k*_)_*j*_ that satisfies the following two conditions (Eqs 5 and 6):

$$\Sigma_{m=s}^{k-1}\left|HAccel\right|\left(t_{m}\right)<1/2\Sigma_{m=s}^{n_{j}}\left|HAccel\right|\left(t_{m}\right)$$ (5)

and

$$\Sigma_{m=k+s}^{n_{j}}\left|HAccel\right|\left(t_{m}\right)\leq 1/2\Sigma_{m=s}^{n_{j}}\left|HAccel\right|\left(t_{m}\right)$$ (6)

The starting indices *s* are thereby set at *t* (*t*_*i*_ − *t*_*dur j*_ / 2) with *i* = 1, ⋯, *n*_*j*_. Analogously to Eq 4, the profiles for the maximum weighted medians of *HSpeed* (*HSpeed_weighted*) against *t*_*dur*_ were calculated from the given sets (Eq 7):

$$HSpeed\_weighted_{max_{j}}=\begin{array}{c} \begin{array}{c} {\text{max}}_{i\in n_{j}}\left(\text{H}Speed\_weighted{\left(t_{i}\right)}_{j}\right) \end{array} \end{array},\;j=1,\cdots ,21$$ (7)

As a result, for each player and game, distinct profiles with values for maximal achieved weighted median *HSpeed* were given to characterize weighted locomotor performance during their games.

#### Nonlinear fittings

To evaluate for regularity and to estimate function parameters for *HSpeed* against *t*_*dur*_, seven nonlinear functions were chosen based on their principal applicability (s. S1 Appendix), as they had logistic or sigmoidal decay characteristics. Prior to the fitting procedure, *t*_*dur*_ was log-transformed for better clarity. For the optimization procedure, Newton-Raphson's method [29] was applied using a stop limit of 300 iterations aimed at 1.0 · 10^-6^ for the relative gradient. The Akaike Information Criterion (AICc) [30] was used as a relevant evaluation criterion to judge fitting quality. Other than the F-statistic, AICc is independent in the comparison of models with a different number of parameters.

#### Statistical analysis

Statistical analyses, model fittings and evaluations were performed using JMP Pro Version 13.1.0 (SAS Institute Inc., Cary, NC, USA). Data are presented as means ± standard deviations unless otherwise indicated. For statistical calculations, a critical significance level of α = 0.05 was assumed. Group comparison considering more than two groups was performed using a nonparametric one-way analysis of variance (Steel-Dwass all-pairs rank-order statistics for non-normally distributed data and data that failed the equal variance test). Where repeated measures occurred in group comparisons (two or more games were analyzed for most of the players), we applied a general linear mixed model [31,32] with participant, gender and total play time as random factors to evaluate for significant differences of the curve fitting parameter results.

## Results

In total, 82 soccer game participations (≥ 30 min) of the 38 players from 3 teams (A, B, C) were analyzed. Total play times, and absolute and relative running distances per minute are given in Table 2. There were no statistically significant differences between the teams in these results.

**Table 2:** Minutes of game participation, total and relative running distances grouped by team.

	Play time (min)		Total running distance (m)		Running distance per minute (m·min^-1^)	
Team	Mean	SD	Mean	SD	Mean	SD
A	85.2	10.5	9953	1238	117.6	11.7
B	81.3	12.6	8323	1024	103.7	13.0
C	86.3	8.7	9283	1046	107.9	10.0
All	84.7	10.4	9340	1271	110.9	12.6

Mixed-model analysis revealed no significance for the random factor "Team" for all measures shown. *SD*: Standard deviation.

**Fig 1:**
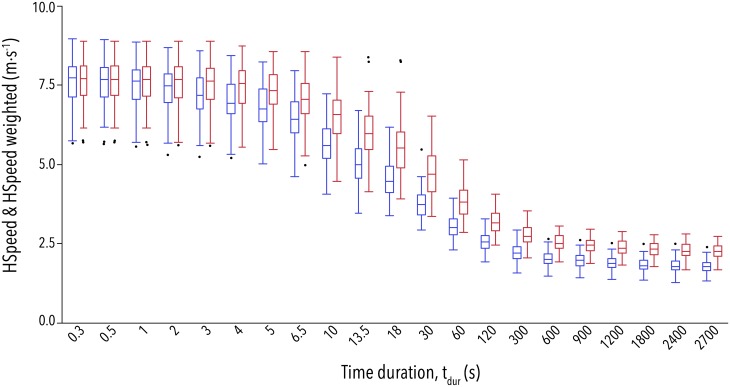
*HSpeed* results overview. Quantile statistics (boxplots) for the maximal horizontal moving speeds (*Hspeed*, *blue*) and HSpeed weighted by acceleration (red) for the 21 calculated time durations (*t*_*dur*,_ from Eq 3).

The quantile statistics of maximum *HSpeed* versus *t*_*dur*_ of all game participations (Fig 1) already represent a characteristically descending sigmoidal shape (S-curve shape with a distinct inflection point) of the relationship.

**Fig 2:**
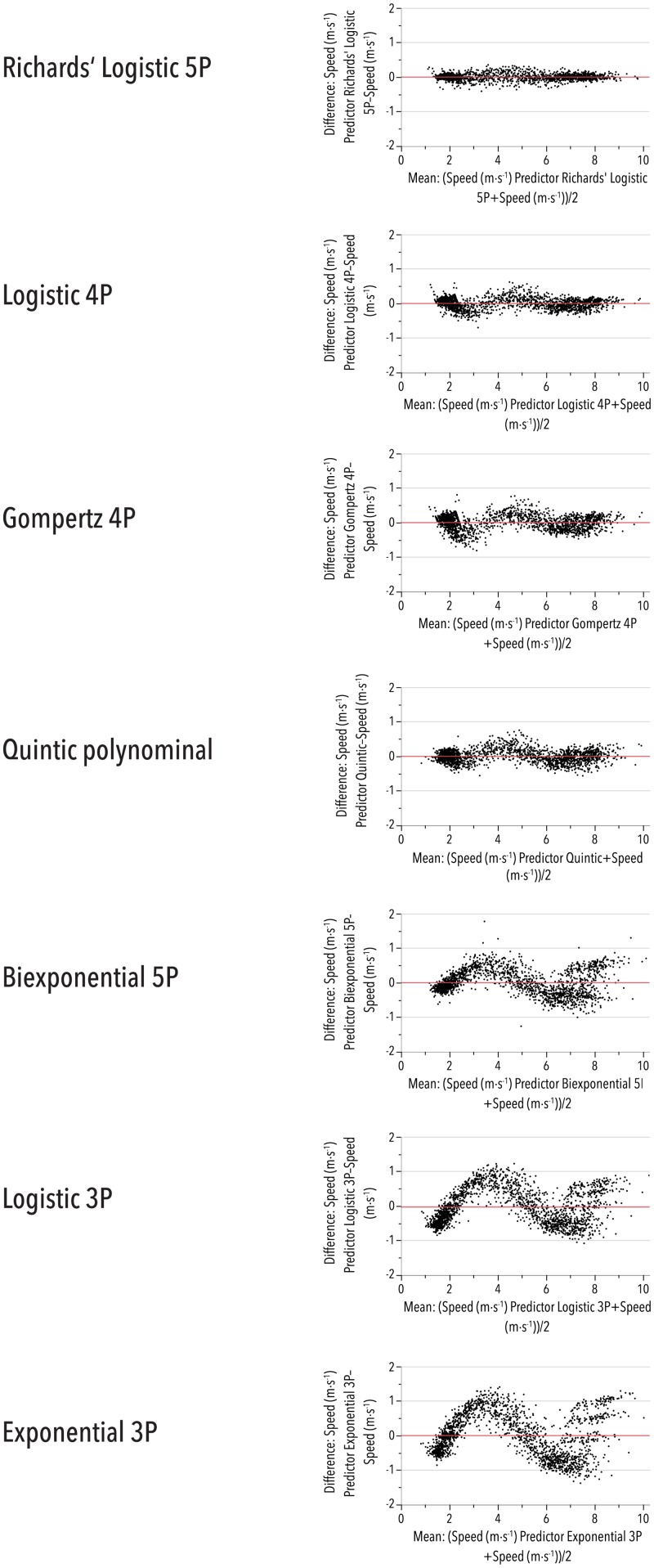
Residuals of the seven evaluated fitting equations. The differences between the measured and the predicted values against the mean between both are shown. Only the Richards' Logistic 5P equation (Eq. 8, S1 Appendix) does not display a systematic deviation from the measurements. For further descriptions of the applied equations, see the S1 Appendix.

During the evaluation of seven formally matching fitting equations for the individual relationships between the log-transformed time duration (*Log*(*t*_*dur*_)) and the related fastest horizontal moving speeds (*HSpeed*), a five-parameter generalized logistic model (adopted from Richards' growth function [33], Eq. 8, S1 Appendix) revealed by far the best fitting quality. Fitting details for all equations are shown in Table 3. Based on individual game participations, root-mean-square error (RMSE) was as low as 0.10 m/s for the best fitting equation without any obvious bias; in contrast, bias was evident for all other models evaluated (Fig 2).

**Table 3:** Overall evaluation of seven models tested to represent the relationship between maximum horizontal moving speed (*HSpeed*; *y*) and time duration of movement (*t*_*dur*_; *x*) for every participant and soccer game tested in the study.

Fittings of $HSpeed_{max_{j}},\;j=1,\cdots ,21$ vs. time duration (*t*_*dur*_)					
	**AICc**	**SSE**	**MSE**	**RMSE** **(m·s**^**-1**^**)**	**R**^**2**^
**1.** Richards (Logistic 5P)	-2265.1	13.9	0.011	0.10	0.999
**2.** Logistic 4P	-759.1	39.4	0.029	0.17	0.996
**3.** Gompertz 4P	560.2	91.6	0.060	0.25	0.992
**4.** Quintic polynominal	583.5	65.6	0.049	0.22	0.994
**5.** Biexponential 5P	3358.8	340.0	0.237	0.49	0.969
**6.** Logistic 3P	3385.5	467.7	0.289	0.54	0.957
**7.** Exponential 3P	4170.4	707.1	0.437	0.66	0.935
Fittings of $HSpeed\_weighted_{max_{j}},\;j=1,\cdots ,21$ vs. time duration (*t*_*dur*_)					
**1.** Richards (Logistic 5P)	-2265.1	13.9	0.011	0.10	0.999
**2.** Logistic 4P	-759.1	39.4	0.029	0.17	0.996
**3.** Gompertz 4P	560.2	91.6	0.060	0.25	0.992
**4.** Quintic polynominal	583.5	65.6	0.049	0.22	0.994
**5.** Biexponential 5P	3358.8	340.0	0.237	0.49	0.969
**6.** Logistic 3P	3385.5	467.7	0.289	0.54	0.957
**7.** Exponential 3P	4170.4	707.1	0.437	0.66	0.935

The models are sorted in ascending order with respect to their AICc. *AICc*: Akaike's Information Criterion, *SSE*: Residual sum of squares error, *MSE*: Mean squared error, *RMSE*: Standard deviation of the residual error, *R*^*2*^: Coefficient of determination. For a detailed description of the formulas applied, see S1 Appendix.

**Fig 3:**
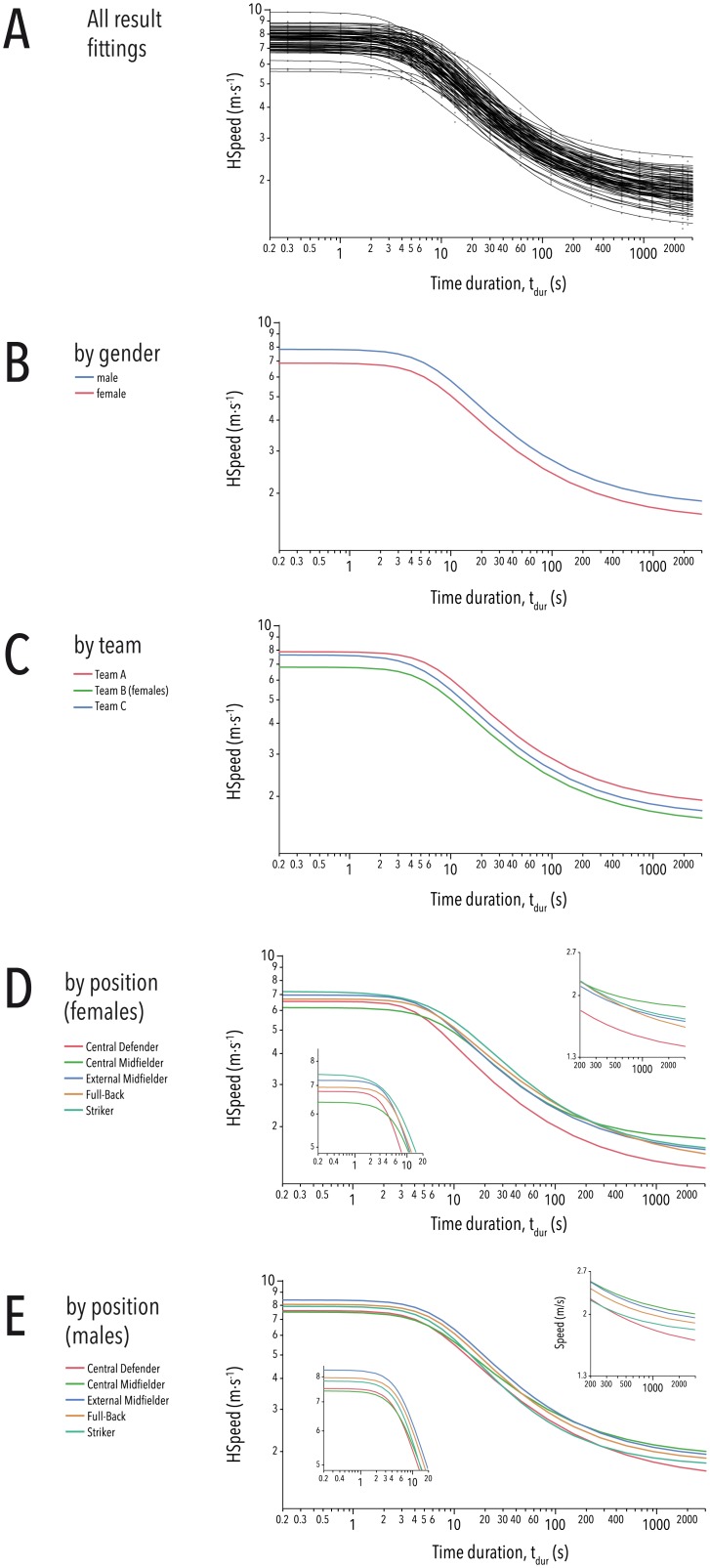
Groups of power-profile curve fittings. (A) All fitting results. (B) Fitting results grouped by gender (blue: male, red: female). (C) Fitting results grouped by team (red: Team A, green: Team B, blue: Team C). (D) Fitting results of all female participants, grouped by position (red: Central Defenders, green: Central Midfielders, blue: External Midfielder, orange: Full-Backs, cyan: Strikers). (E) Fitting results of all male participants, grouped by position (red: Central Defenders, green: Central Midfielders, blue: External Midfielder, orange: Full-Backs, cyan: Strikers). *HSpeed* was log-transformed for better clarity in all cases. The close-ups in (D) and (E) show the upper and the lower part of the fittings, respectively.

The range of parameter values for Eq. 8, S1 Appendix regarding each player's position, sex or team are given in Table 4. Remarkably, neither decreasing rate (*e*^*a*^) nor power (*f*) showed any significant differences when comparing factors of sex, team or position, while inflection point (*e*^*b*^), lower (*c*) and upper asymptotes (*d*) differed depending on position, sex, and team. In particular, Central Defenders and Central Midfielders showed significantly lower values for top speed ("Sprint excess", *d*) than did all other game positions. Similarly, Central Midfielders showed significantly higher values for *c*, or long term running speed ("Critical speed"), while Central Defenders had the lowest values for *c*. Fig 3A–3E gives a graphical overview of the fitted functions for all participants ordered by their position, gender, or team.

**Table 4:** Mean ± SD values for the parameters found with fittings of Eq. 8, S1 Appendix) to the relationship between time duration of movement (*t*_*dur*_) and maximum horizontal moving speed (*HSpeed*_*max*_) for all players.

		Decrease rate (e^a^) m·s^-2^		Inflection point (e^b^) s			Lower asymptote (c) m·s^-1^				Upper asymptote (d) m·s^-1^				Power (f) –	
*All*																
		0.103 ± 0.079		6.92 ± 2.74			1.73 ± 0.24				7.62 ± 0.69				0.32 ± 0.17	
*By Position*																
	Central Defender	0.090 ± 0.069	A	5.54 ± 1.92	A		1.48 ± 0.18	A			7.35 ± 0.49	A	B		0.26 ± 0.10	A
	Central Midfielder	0.113 ± 0.077	A	7.79 ± 3.84		B	1.91 ± 0.17		B		7.21 ± 0.71		B		0.35 ± 0.21	A
	External Midfielder	0.095 ± 0.080	A	6.75 ± 2.30	A	B	1.77 ± 0.25			C	7.97 ± 0.73			C	0.30 ± 0.16	A
	Full-Back	0.100 ± 0.070	A	6.99 ± 2.18	A	B	1.72 ± 0.19			C	7.76 ± 0.61	A		C	0.30 ± 0.12	A
	Striker	0.123 ± 0.108	A	7.28 ± 2.52		B	1.68 ± 0.17			C	7.77 ± 0.44	A	B	C	0.35 ± 0.20	A
*By Gender*																
	Female	0.100 ± 0.078	A	6.99 ± 3.52	A		1.58 ± 0.20	A			6.88 ± 0.53	A			0.31 ± 0.19	A
	Male	0.104 ± 0.080	A	6.89 ± 2.74	A		1.79 ± 0.23		B		7.87 ± 0.55		B		0.32 ± 0.19	A
*By Team*																
	“Team A”	0.093 ± 0.078	A	7.13 ± 2.40	A		1.85 ± 0.25	A			7.95 ± 0.48	A			0.29 ± 0.16	A
	“Team B”	0.100 ± 0.078	A	6.99 ± 3.52	A		1.58 ± 0.20		B		6.88 ± 0.53		B		0.31 ± 0.19	A
	“Team C”	0.116 ± 0.084	A	6.65 ± 2.54	A		1.70 ± 0.18	A	B		7.78 ± 0.61	A			0.33 ± 0.16	A

Different letters indicate significant differences after application of a mixed model analysis with repeated structure.

To better reflect the match-specific endurance requirements of soccer players, we also applied profile fittings using Eq. 8 (S1 Appendix) to *HSpeed* after weighting the median values by each participant’s acceleration values (Eq 7). The fitting results for this alternative approach are shown in Table 5. We assumed that weighing by acceleration would lead to better representations of the discontinuous locomotor events that occur during soccer matches. However, this type of analysis did not further discriminate players by position, sex or team.

**Table 5:** Mean ± SD values for the parameters found when Eq. 8 (S1 Appendix) was used to model the relationship between time duration of movement (*t*_*dur*_) and the weighted maxima of horizontal moving speed (*HSpeed_weighted*_*max*_, Eq 7) for all players.

		Decrease rate (e^a^) m·s^-2^		Inflection point (e^b^) s			Lower asymptote (c) m·s^-1^				Upper asymptote (d) m·s^-1^				Power (f) –		
*All*																	
		0.066 ± 0.076		8.98 ± 4.06			2.08 ± 0.32				7.62 ± 0.67				0.20 ± 0.17		
*By Position*																	
	Central Defender	0.079 ± 0.083	A	8.12 ± 2.67	A		1.84 ± 0.27	A			7.37 ± 0.53	A	B		0.20 ± 0.13	A	B
	Central Midfielder	0.089 ± 0.070	A	9.92 ± 5.02		B	2.18 ± 0.28		B		7.26 ± 0.73		B		0.26 ± 0.19	A	
	External Midfielder	0.098 ± 0.116	A	9.88 ± 4.16	A	B	2.15 ± 0.33		B	C	8.02 ± 0.64			C	0.21 ± 0.19	A	B
	Full-Back	0.057 ± 0.094	A	7.96 ± 2.94	A	B	2.05 ± 0.30	A		C	7.79 ± 0.62	A		C	0.14 ± 0.13	A	B
	Striker	0.089 ± 0.149	A	7.71 ± 4.48	A	B	2.08 ± 0.32	A	B	C	7.82 ± 0.46	A	B	C	0.11 ± 0.09		B
*By Gender*																	
	Female	0.050 ± 0.057	A	8.13 ± 3.20	A		1.84 ± 0.27	A			6.89 ± 0.54	A			0.22 ± 0.27	A	
	Male	0.072 ± 0.082	A	9.28 ± 4.31	A		2.16 ± 0.30		B		7.87 ± 0.51		B		0.32 ± 0.48	A	
*By Team*																	
	“Team A”	0.058 ± 0.076	A	9.80 ± 4.71	A		2.22 ± 0.32	A			7.99 ± 0.48	A			0.19 ± 0.19	A	
	“Team B”	0.050 ± 0.057	A	8.13 ± 3.20	A		1.84 ± 0.27		B		6.89 ± 0.54		B		0.17 ± 0.13	A	
	“Team C”	0.088 ± 0.087	A	8.61 ± 3.72	A		2.15 ± 0.24	A			7.73 ± 0.52	A			0.23 ± 0.15		B

Different letters indicate significant differences after application of a mixed model analysis with repeated structure.

**Fig 4:**
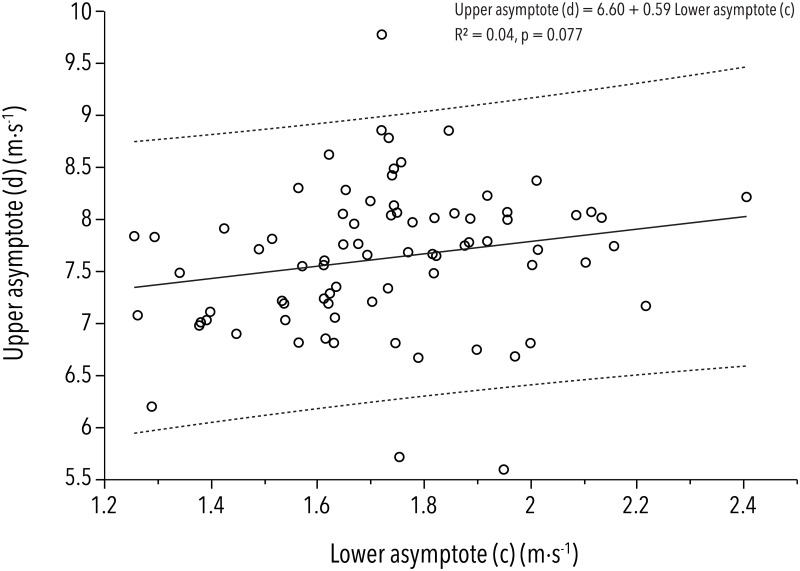
Independence of the lower against the upper asymptotes. A linear (Pearson) correlation between parameters *c* and *d* from all individual measurements is given (r^2^ = 0.04).

As we used repeated measurements from several players, we performed a standard least squares analysis with the player's identity as a random effect. This analysis yielded r^2^-values (in decreasing order) for the upper asymptote (*d*) r^2^ = 0.79 (weighted by acceleration r^2^ = 0.75), lower asymptote (*c*) r^2^ = 0.64 (weighted by acceleration r^2^ = 0.57), inflection point (*b*) r^2^ = 0.57 (weighted by acceleration r^2^ = 0.11), decrease rate (*a*) r^2^ = 0.29 (weighted by acceleration r^2^ = 0.09), and power (*f*) r^2^ = 0.02 (weighted by acceleration r^2^ = 0.11). Furthermore, the correlation analysis shown in Fig 4 illustrates statistical independence between the first two variables, *c* and *d*.

## Discussion

### Main findings

In this study, we showed for the first time that a sigmoidal decaying function describes the numerical relationship between horizontal moving speed and the log-transformed time duration of running for soccer players during a match. A five-parameter logistic decay function derived from the so-called Richard's Logistic equation fit this relationship with almost zero systematic bias. Using this function, the upper and lower asymptotes and the points of inflection could discriminate the players’ characteristics and positions. The remaining parameters "power" (*f*) and "decrease rate" (*a*) of *HSpeed* were not different between participants and tests, meaning that there was a common and reliable curve shape in every case.

### Methodological aspects

We used commercially available GPS sensors with only limited technical documentation in this study. The reliability of GPS-measured velocities has been reported to depend on many factors including sampling frequency and, especially, moving velocity (increased velocity reduces reliability) [34]. The manufacturers state that an integrated Kalman-type filter [24,35] and sensor-fusion algorithms using an accelerometer, a magnetometer, and a gyroscope "*to adjust the reference frame of the sensor to the horizontal Earth's surface with a frequency of 10Hz*" are used for the sensors [36]. An internal validation against a video analysis system (Immotio, Amsterdam, NL) resulted in mean differences of only 0.1 to 0.3 m·s^-1^ at the 95% confidence level [36], indicating excellent validity of the applied sensor system. Also, the particular profiles analyzed in this study yielded physiologically credible results. For example, for 30 m, the fastest sprints ranged from 3.46 s to 5.91 s (flying), which are overall plausible results for the tested selection of players and also compare favorably to the reference data of other researchers [37,38]. The fitting procedure applied further minimizes noise from the measurements of *HSpeed*, because separate deviating data points are adjusted to the curve's function.

Maximum acceleration values measured by wearable devices can yield erroneous results due to passive impacts or tackles to the body [39]. However, such collision-related impulses only last for fractions of a second, and seldom affect standing or slowly moving players (the only conditions that could cause positive, forward-directed acceleration). From the raw accelerometry data, a maximum positive outlier value within a time duration of 0.3 sec was determined at 8.6 m·s^-2^ (mean: 6.2 ± 0.9 m·s^-2^). At 1.0 sec, for comparison, this value decreased markedly to an overall maximum of 7.1 m·s^-2^ (mean: 5.1 ± 0.7 m·s^-2^, p < 0.0001). This is why low-pass filtering is recommended to improve the validity of accelerometers in team sports [5]. However, *HSpeed* profiles are most likely not affected by this phenomenon of passive impact, because acceleration must be effective for a longer time to induce the maximum *HSpeed* values that were observed in this study. Otherwise, reliability of acceleration data from trunk mounted systems is generally poor, especially at higher levels of acceleration [5]. Thus, weighting the profiles with acceleration did not improve the characterization of the players in this study (Tables 4 and 5). The search for alternative means of determining the factor *agility* is a promising area for future research.

Isolated tests of sprint running performance tend to have high variability and low reliability [40]. However, maximum sprint speeds are important factors in the functional assessment of players, and are specifically targeted during training interventions in soccer [41,42]. Similar arguments can be used for mid- and long-term running endurance performance [43,44]. The methodology that we describe here allows direct assessment of these performances. With each reiteration during a player's season, particularly at the highest intensity as is achieved during competitions, the characterization of the player will be more reliable. Thus, the approach presented here can lead to much more comprehensive information about individual performance than other conventional performance testing methods that use out-of-competition sprint time analysis or laboratory exercise testing.

### Curve shape of HSpeed against t_dur_

The relationship between maximum power output and time has been discussed primarily in the field of occupational physiology. At first, Monod and Scherrer proposed the term "*critical power [*…*] from the notions of maximum work and maximum time of work"* [45]. This 'critical' power (W_lim_) is one parameter of several alternative non-linear functions that are all characterized by an asymptotic approximation of power output at [45] or below [46] *t*_*lim*_ = 0. Since that introduction, intense and diverse discussions have taken place regarding the best numerical interrelationship between power output and time [47], with the aim of achieving interpretable parameters for performance diagnostics and participant characterization, especially in endurance sports [48–50]. It turns out that experimental approaches to obtaining complete power-to-duration profiles are a challenging task: several maximum exercise tests in the relevant time domains are needed to form the basis for comprehensive curve fitting. This makes routine applications of power-to-duration analyses virtually impossible, and might be the major reason why modeling approaches have been developed to predict the power duration relationship from ordinary test procedures [51,52].

Other studies have determined a 'critical speed' from running instead of work measurements [53,54] based on the assumption that running velocity is linearly correlated to energy expenditure. This is (approximately) true when running at steady speeds with small accelerations [55]. However, our study used data from intermittent, not steady, running. That is why the lower asymptotes (“Critical speed”) are at very slow speeds (2.0 to 2.5 m·s^-1^), although most of the players reached their metabolic limits. To compensate for this, we tried the weighting by acceleration, which caused interval type variations of HSpeed to be weighted higher. In spite of this, the sigmoidal characteristics of the performance/duration relationship remained the same (s. Fig 1). Further investigation is needed to determine whether the model that fit our data would also apply to continuous running events over short intervals.

Profiles with intermittent maximal sprinting performances might be mathematically much more complex and hypothetically not describable with a simple or bi- exponential function. In addition to pure energy expenditure, neuromuscular, power-related and coordinative factors determine maximum running speeds over short [56] to ultra-short (< 2 sec) time durations [5,57]. Most importantly, these fast ultra-short sprints could be a prerequisite for successful participation in team sports [27,58,59].

Furthermore, our observations are in line with previous theoretical discussion by Bundle and Weyand [56] who claimed that there were specific limitations to all-out sprint exercise performances below 60-s duration due to these performances were determined by not only metabolic energy availability but also by musculoskeletal force application. The upper asymptote and flattening of the performances below 60 s, reported for the first time in this paper, are probably related to “non-metabolic” performance-limiting factors, as proposed by Bundle and Weyand.

### Conclusions

**Fig 5:**
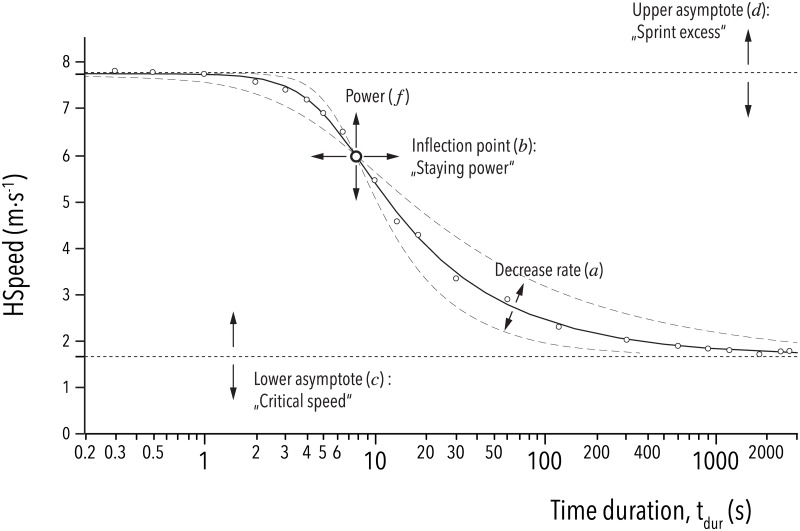
Graphical description of the fitting parameters. The five parameters within the favored fitting model for the relationship between *HSpeed* and *t*_*dur*_ (Eq. 8, S1 Appendix). Small open circle symbols (o) are from real results for one participant in one game.

This study shows for the first time empirical proof that the relationship between maximum running speed and *t*_*dur*_ is better represented with a sigmoidal curve than with a simple exponential function. This type of curve shape was found in every game participation in this study, with no exceptions. A five-parameter function (s. S1 Appendix, Eq. 8; Fig 5) was sufficient to fit every profile without any bias. Furthermore, this sigmoidal curve shape varied individually in terms of upper ("Sprint excess") and lower ("Critical speed") asymptotes, and by t_dur_ of the inflection point (staying power at maximum sprint speed). The other two parameters of this function, power and decreasing rate, did not vary individually, emphasizing the ubiquitousness of the described curve shape. Further work will be needed to validate the model by the modification of selective parameters over time and after certain types of training.

## Supporting information

S1 Appendix (PDF)
