# Supplementary material for: The relationship between movement speed and duration during soccer matches
Source: PLoS One. 2017 Jul 25;12(7):e0181781. doi: 10.1371/journal.pone.0181781 (PMC5526535; doi:10.1371/journal.pone.0181781)
Supplement: S1 Appendix — (PDF) [file pone.0181781.s001.pdf]

Supporting Information

S1 Appendix.

In the following, the seven equations evaluated by nonlinear fittings for *HSpeed* against t_{dur} are described.

1. A modified Richards' logistic five-parameter function, also known as the generalized logistic function (Eq. 8, *Richards' Logistic 5P*):

$$HSpeed(t_{dur}) = c + \frac{(d-c)}{(1+e^{-a\cdot(Log(t_{dur})-b)})^{f}} \tag{8}$$

whereby a is the decreasing rate of $HSpeed$ (per second), and b describes the inflection point of the sigmoidal curve representing t_{dur} with the maximum changing rate of $HSpeed.$ c is the lower asymptote (corresponding to the speed performed during a maximum amount of time, analogously to the so-called "critical speed"), d the upper asymptote (corresponding to maximum running speed achieved during short periods of time, or "Sprint excess"), f is the power of the term, t_{dur} is the related time duration, and $HSpeed$ is the corresponding maximum running speed performed at the related t_{dur}.

2. A generalized four-parameter logistic function without the power component (Eq. 9), *Logistic 4P*):

$$HSpeed(t_{dur}) = c + \frac{(d-c)}{(1+e^{-a\cdot(Log(t_{dur})-b)})} \tag{9}$$

The parameters in Eq. 9 are the same as those in Eq. 8, except power (f) is set to 1.0.

3. A three-parameter logistic function with the lower asymptote at 0 (Eq. 10, *Logistic 3P*):

$$HSpeed(td) = \frac{\mathrm{d}}{(1+e^{-a\cdot(Log(t_{dur})-b)})} \tag{10}$$

4. A quintic polynomial function (fifth-order polynomial) (Eq. 11, *Quintic polynomial*):

$$HSpeed(t_{dur}) = a + b \cdot Log(t_{dur})^5 + c \cdot Log(t_{dur})^4 + d \cdot Log(t_{dur})^3 + f \cdot Log(t_{dur})^2 + g \cdot Log(t_{dur}) \quad (11)$$

5. A bi-exponential function with five parameters (Eq. *12*, *Biexponential 5P*):

$$HSpeed(t_{dur}) = a + b \cdot e^{-c \cdot Log(t_{dur})} + d \cdot e^{-f \cdot Log(t_{dur})} \quad (12)$$

Hereby, *a* is the lower asymptote, *b* and *d* are the scales of two exponentials, and *c* and *f* are the decay rates of the two partially exponential functions.

6. A four-parametric logistic Gompertz' function (Eq. 13, *Gompertz 4P*):

$$HSpeed(t_{dur}) = a + (b - a) \cdot \mathrm{e}^{-e^{-c \cdot (Log(t_{dur}) - d)}} \quad (13)$$

Here, *a* is the lower and *b* is the upper asymptote (maximal running speed in long-term and maximum overall running speed), *c* is the decrease rate of $HSpeed$, and *d* the inflection point of the curve.

7. A three-parametric exponential decay function (Eq. 14, Exponential *3P*):

$$HSpeed(t_{dur}) = \frac{c}{(1 + e^{-a \cdot (Log(t_{dur}) - b)})} \quad (14)$$

Here, *a* is the decrease rate of $HSpeed$, *b* is the inflection point of the curve, and *c* is the lower asymptote.
